# Supplementary material for: Implementation status of national tuberculosis infection control guidelines in Bangladeshi hospitals
Source: PLoS One. 2021 Feb 16;16(2):e0246923. doi: 10.1371/journal.pone.0246923 (PMC7886225; doi:10.1371/journal.pone.0246923)
Supplement: S1 File — (DOCX) [file pone.0246923.s001.docx]

Supporting Information-1

**TB Infection Control measures to be implemented** (facility assessment tool):

| **TB Infection Control measures** | **Yes** | **No** | **Comments** |
| --- | --- | --- | --- |
| **Managerial** |  | |  |
| Coordinating body or responsible  person in place |  |  |  |
| Surveillance and assessment of TB  among health care workers |  |  |  |
| TB infection control plan in place |  |  |  |
| Staff trained in TB infection control |  |  |  |
| Advocacy, communication and social mobilization |  |  |  |
| Monitoring and evaluation  conducted |  |  |  |
| Operational research |  |  |  |
| **Administrative** |  |  |  |
| Triage |  |  |  |
| Separation / cohorting |  |  |  |
| Cough etiquette |  |  |  |
| Expedient service delivery |  |  |  |
| Prevention and care package for health care workers |  |  |  |
| **Environmental** |  |  |  |
| Natural and/or mechanical  ventilation |  |  |  |
| Fans |  |  |  |
| Ultraviolet Germicidal Irradiation |  |  |  |
| **Personal protective equipment** |  |  |  |
| Respirators available for staff |  |  |  |
| Fit testing and/or fit check |  |  |  |
