# Supplementary material for: Implementation status of national tuberculosis infection control guidelines in Bangladeshi hospitals
Source: PLoS One. 2021 Feb 16;16(2):e0246923. doi: 10.1371/journal.pone.0246923 (PMC7886225; doi:10.1371/journal.pone.0246923)
Supplement: S2 File — (DOCX) [file pone.0246923.s002.docx]

Supporting Information-2

**COREQ table:**

|  |  |  |  |
| --- | --- | --- | --- |
| **Table: Consolidated criteria for reporting qualitative studies (COREQ): 32-item checklist** | | | |
|  | **No** | **Item** | **Description** |
|  | **Domain 1: Research team and reflexivity** | | |
|  | **Personal Characteristics** | |  |
|  | 1 | Interviewer/facilitator | One of the first authors and three co-authors |
|  | 2 | Credentials | Researchers with BSc, MBA, MSS, MBBS, MPH, PhD degrees. |
|  | 3 | Occupation | All are Researcher by profession |
|  | 4 | Gender | Male (five) and Female (three) |
|  | 5 | Experience and training | Researchers have: long time experience on TB field and directly working with NTP, and also have work experience of TB infection control studies, three of them are clinicians, one having international training on infection control |
|  | **Relationship with participants** | |  |
|  | 6 | Relationship established | The team has around 12 years working relationship with the hospital healthcare workers. |
|  | 7 | Participants Knowledge of the interviewer | Participants were well informed about the study purpose, objectives, methodology prior to data collection |
|  | 8 | Interviewer characteristics | Interviewer were not biased, had optimum knowledge on the research topic |
|  | **Domain 2: Study design** | | |
|  | **Theoretical framework** | |  |
|  | 9 | Methodological orientation and Theory | Grounded theory |
|  | **Participant selection** | |  |
|  | 10 | Sampling | Convenient and purposive |
|  | 11 | Method of approach | Face to face |
|  | 12 | Sample size | 59 |
|  | 13 | Non participation | Three (two senior physicians and one hospital director), as they were not able to manage their time for interviews |
|  | **Settings** | |  |
|  | 14 | Setting of data collection | Participants workplace (preferred by participants) |
|  | 15 | Presence of non-participants | Presence of non-participants was controlled. |
|  | 16 | Description of sample | Descriptive data |
|  | **Data collection** | |  |
|  | 17 | Interview guide | Followed interview guidelines, also provided to the respondents if asked, the guidelines were pilot tested |
|  | 18 | Repeat interviews | No repeat interviews needed |
|  | 19 | Audio/visual recording | Audio recording used during data collection |
|  | 20 | Field notes | During and after the interviews, field notes were documented |
|  | 21 | Duration | Average duration of interviews was 42 minutes |
|  | 22 | Data saturation | Data saturation discussed and assed before completing data collection |
|  | 23 | Transcripts returned | No, transcriptions were not returned to the participants for comments or corrections individually but summary findings was shared with the participants and they agreed on the findings. |
|  | **Domain 3: Analysis and findings** | | |
|  | **Data analysis** | |  |
|  | 24 | Number of data coders | Three |
|  | 25 | Description of the coding tree | Coding information described in the manuscript |
|  | 26 | Derivation of themes | Themes identified in advance according to the four component of the TB-IPC guideline |
|  | 27 | Software | We did manual data analysis, software was not used |
|  | 28 | Participant Checking | Participants provided general positive feedback on the summary data shared with the facilities |
|  | **Reporting** | |  |
|  | 29 | Quotation presented | Yes, participant’s quotation was presented. Quotations were identified but not by the participant number, it was mentioned about the characteristics of the respondent. |
|  | 30 | Data and findings consistent | Data presented and findings are consistent |
|  | 31 | Clarity of major themes | Major themes were clearly presented in the findings |
|  | 32 | Clarity of minor themes | Minor themes also discussed |
