# Supplementary material for: Implementation status of national tuberculosis infection control guidelines in Bangladeshi hospitals
Source: PLoS One. 2021 Feb 16;16(2):e0246923. doi: 10.1371/journal.pone.0246923 (PMC7886225; doi:10.1371/journal.pone.0246923)
Supplement: Attachment — Submitted filename: Reviewers comments.docx [file pone.0246923.s003.docx]

19^th^ September, 2020

The Editor,

PLoS One

Dear Sir,

Thank you for giving me the opportunity to review this interesting manuscript titled “Implementation status of national tuberculosis infection control guidelines in Bangladeshi hospitals” The study assessed the implementation of the TB IPC healthcare measures in health settings, Bangladesh using mixed methods. The topic is topical and relevant. Please see below some comments regarding issues that should be addressed

**In the introduction section:**

Line 53: The sentence ‘TB mortality rate was 36 per 100,000’ is not complete, Mortality rate globally or locally?

Line 58: Are all healthcare workers exposed or those working in TB clinics?

**In the methodology section:** - It will be nice to know the number of persons that were involved in the data collection and the geographical distribution of the healthcare settings used for this study across Bangladesh. (Lines 84-87).

In what language was the KII conducted?

Ethics: The name of the IRB and the approval number should be provided. (lines 112-114)

**In the Results section**: Table 2: It will be nice to know which of the distribution of the hospitals either as TB specialty hospitals or tertiary care hospitals instead of numbering.

How many of the participating physicians were directors of the hospitals, heads of medicine and senior physicians?(Line 117)

Line 128: ‘only laboratory equipment’s monitoring in terms of functionality were in place in four health settings’--- were these hospital TB specialty hospitals or tertiary care hospitals?

Operational research: the category of the only centre that carried out operational research should be stated. (Line 176)

Line 216: How many of the TB specialty hospitals are non-governmental?

Line 228: without categorizing the hospital settings in table 2, the statement ‘All the tertiary care hospitals lacked UVGI (Table 2)’ is not justifiable.

**References:**

Number 7: List the names of five authors before et al.

Number 21: The journal name, issue no, volume if any and page numbers should be provided.

In addition, the whole manuscript should be edited and grammatical errors addressed for example not limited to;

Line 118-119: ‘The mean age of the participants was 45 years with mean job duration **of** 10 years’

Line 313-314: ‘To enable the health system for better implementation of TB IPC guideline, the national TB IPC guidelines should be introduced in all health settings dealing **with** TB cases…..’

Line 319: ‘guidelines among the hospitals that resulted **in** poor adherence to the TB IPC measures…..’

Regards

Dr. Yetunde Kuyinu
